# Inclusion of Sorghum in *Cyprinus carpio* L. Diet: Effects on Growth, Flesh Quality, Microbiota, and Oxidative Status

**DOI:** 10.3390/ani14111549

**Published:** 2024-05-24

**Authors:** Cristian-Alin Barbacariu, Gabriela Dumitru, Cristina Mihaela Rimbu, Cristina Elena Horhogea, Lenuța Dîrvariu, Elena Todirașcu-Ciornea, Dana Andreea Șerban, Marian Burducea

**Affiliations:** 1 Research and Development Station for Aquaculture and Aquatic Ecology, “Alexandru Ioan Cuza” University, Carol I, 20A, 700505 Iasi, Romania; alin.barbacariu@uaic.ro (C.-A.B.); lus22grigorica@yahoo.com (L.D.); dana.serban@uaic.ro (D.A.Ș.); 2 Faculty of Biology, “Alexandru Ioan Cuza” University, Carol I, 20A, 700505 Iasi, Romania; ciornea@uaic.ro; 3 Department of Public Health, Faculty of Veterinary Medicine, University of Life Sciences ‘’Ion Ionescu de la Brad’’ Iaşi, Mihail Sadoveanu Alley 6-8, 700490 Iasi, Romania; cristina.rimbu@iuls.ro (C.M.R.);; 4 Faculty of Food and Animal Sciences, University of Life Sciences “Ion Ionescu de la Brad” Iaşi, Mihail Sadoveanu Alley 6-8, 700490 Iasi, Romania

**Keywords:** sorghum, common carp, aquaculture, growth performance, hematology, intestinal microbiota, oxidative stress

## Abstract

**Simple Summary:**

Sorghum is a nutritious crop that thrives in diverse climates and can withstand harsh growing conditions, making it an attractive option for feeding farmed fish in various regions around the world. In this study, the effects of different sorghum-containing diets on carp were tested, revealing a reduction in growth rates, notable changes in flesh protein content, and significant effects on intestinal microbiota and oxidative stress. This indicates sorghum’s potential health and metabolic impacts on *Cyprinus carpio*.

**Abstract:**

This study investigates the impact of including sorghum in the diet of the common carp (*Cyprinus carpio*) on its growth, blood parameters, meat composition, intestinal microbiota, and oxidative stress. Experimental diets with varying sorghum content (0%—V0 or control, 10%—V1, 20%—V2, and 30%—V3) were administered to carp weighing 43 g initially. Notably, in the 30% variant, sorghum entirely replaced corn and barley in the diet. Chemical analysis of sorghum unveiled a protein content of 14% and a fat content of 3.9%. Sorghum inclusion led to a decline in final body weight and weight gain, particularly notable in the V3 group with 30% sorghum. However, other physiological parameters, such as feed conversion ratio, specific growth rate, and organ indices, remained unaffected. Protein and salt content in carp flesh increased with higher sorghum inclusion levels, while hematological parameters showed minimal variations. Analysis of the intestinal microbiota revealed increases in both aerobic and anaerobic bacterial populations with sorghum inclusion. Furthermore, sorghum concentration inversely correlated with glutathione levels and positively correlated with malondialdehyde content, indicating a disruption of antioxidant defense mechanisms and elevated oxidative stress.

## 1. Introduction

Aquaculture plays a pivotal role in meeting the global demand for food and nutrition, being the fastest growing food industry sector. According to the latest report from the Food and Agriculture Organization (FAO) released in 2022 [1], aquaculture contributed 122.6 million tons out of the total 214 million tons of global fishery and aquaculture production in 2020, valued at USD 281.5 billion. Looking ahead, the FAO projects a 14 percent increase in fish production by 2030, with aquaculture playing a crucial role in this growth trajectory [1]. The sustained expansion of aquaculture will contribute significantly to achieving Sustainable Development Goals (SDGs), particularly SDG 2 (Zero Hunger), SDG 14 (Life Below Water), and SDG 8 (Decent Work and Economic Growth). However, the sector faces significant challenges, particularly related to feed, which represents over 50% of production costs [1]. The challenge regarding feeding stems from the rising costs and scarcity of traditional ingredients like fish meal and fish oil, necessitating the exploration of sustainable alternatives. In this context, identifying and integrating alternative ingredients into aquafeed formulations becomes increasingly crucial. This not only addresses economic concerns, but also aligns with sustainability goals by reducing reliance on finite marine resources.

The chemical composition of sorghum (*Sorghum bicolor*) is similar to that of other cereals used in aquaculture (such as corn, wheat, barley, etc.), with approximately 11.4% protein, 3.3% fat, and 1.9% fiber [2]. One of the beneficial features of sorghum is its ability to be cultivated in semi-arid areas, with the United States being the world’s leading producer, contributing to around 15% of total production [3]. The main advantages of using sorghum in fish feed include high antioxidant content, nutrient source, non GMO status, sustainability, and significant global production [4,5,6,7]. However, challenges in using sorghum include its lower protein content (similar to other cereals), reduced starch digestibility, the presence of antinutritional factors, lysine and threonine deficiencies, and limited studies on its application in fish feeding [4,5,6,7]. Key antinutritional factors in sorghum include trypsin inhibitors, amylase inhibitors, tannins, and phytic acid [8]. 

In addition to its various advantages and challenges in aquaculture, sorghum’s cultivation in semi-arid areas faces increasing pressures due to climate change. These regions are experiencing heightened aridity, exacerbated by factors such as deforestation and desertification, affecting the yield and quality of sorghum crops [9]. Furthermore, amidst the global challenges posed by the COVID-19 pandemic and geopolitical tensions, including the Ukraine conflict, contributing to a surge in cereal prices, the exploration of alternatives gains significance [10]. Although Europe, including Romania, is not among the top sorghum-producing regions globally, its yields are quite impressive. According to Popescu et al. [11], in 2017, the EU produced 1.18% of the world sorghum output on only 0.12% of the global sorghum cultivation area. Romania contributed 7.18% to the EU’s total production. With a yield per surface unit of 3879 kg/ha, Romania surpassed the world average by 2.71 times. Recognizing the potential benefits of sorghum cultivation and the urgent need to address climate change, EU policies are geared towards encouraging the expansion of cultivated areas and production. 

The common carp (*Cyprinus carpio* L.) is the most important species cultivated in Romania and is considered a traditional species in many European countries such as Hungary, Poland, the Czech Republic, Bulgaria, Slovenia, and Lithuania, with its production reaching 36,403 tons in 2021 in the mentioned countries, according to Eurostat [3]. Carp-farming systems in Romania are typically semi-intensive, utilizing earthen ponds and incorporating cereals and oilseeds in feed formulations. The main issues related to the use of plant-based ingredients stem from the presence of antinutritional factors and their low digestibility. In this context, D’mello [12] demonstrated that sorghum digestibility in carp is roughly similar to that of rye in terms of lipids and slightly lower in terms of protein. Degani highlighted in his study that the protein digestibility of sorghum in carp is 71.86%, while lipid digestibility is 76.71% [13]. 

Considering the traditional aquaculture nature of carp farming, farms engaging in this activity also play a role in maintaining these practices, contributing to the creation of local ecosystem services [14]. The relevance of sustainable feeds for carp farming lies in their crucial role in fostering sustainable aquaculture practices, which are essential for preserving local ecosystem services. In animal farming (pigs, poultry, ruminants), sorghum is commonly used and usually replaces corn [15]. A study by Anderolu [16] demonstrated the possibility of replacing corn with sorghum in ratios of up to 50% without negative effects on growth or health in the African catfish (*Clarias gariepinus*). Another study showed the potential replacement of cassava with sorghum in ratios of up to 20% in *Pangasius pangasius* [17]. 

In light of these considerations, this study aims to assess the viability of sorghum as a determinant ingredient in the common carp’s diet, with a specific focus on determining the optimal inclusion levels and investigating its effects on growth performance, blood parameters, meat quality, intestinal microbiota, and oxidative stress.

## 2. Materials and Methods

### 2.1. Fish Farming and Management

In the spring of 2023, at the Research and Development Station for Aquaculture and Aquatic Ecology, directed natural reproduction was conducted in an earthen pond using carp broodstock from the station. A total of 120 carp fingerlings were selected from a pool of 8000 individuals, with an initial weight of 43 g. The fish were transferred to the station’s recirculating aquaculture system (RAS) and acclimated for two weeks before the start of the experiment. The RAS was equipped with water tanks, drainage pipes, feeding systems, a mechanical filter, a biological filter, a UV filter, recirculation pumps, automation, O_2_ sensors, temperature sensors, and pH sensors. The feeding trial had a duration of six weeks, beginning on 7 August and ending on 15 September. Throughout the trial, the water temperature started at 25.6 °C and decreased to 21.7 °C by the end (Table 1). Daily measurements included dissolved oxygen and temperature, while pH, ammonia, nitrites, nitrates, and phosphorus were measured weekly. Water parameters (ammonia, nitrites, nitrates, phosphates) were measured using the Hanna Iris HI801 Spectrophotometer and Hanna reagent kits (Hanna Instruments, Salaj, Romania), Hach HQ30d (Hach Company, Loveland, CO, USA) (dissolved oxygen), and Hach HQ11d (Hach Company) (pH and conductivity). Water parameters are presented in Table 1.

### 2.2. Experimental Diets

Four feed diets were created, with the proportion of sorghum varying as follows: control—0%, V1—10%, V2—20%, V3—30%. The control diet consisted of sunflower meal 20%, green peas 20%, corn 15%, barley 12%, corn DDGS 11%, fish meal 20%, and sunflower oil 2%, without sorghum. The ingredients used (sunflower meal, green peas, corn, barley, corn DDGS, fish meal, sunflower oil, and sorghum) were specific to carp diets and originated from Romania, except for the fishmeal. The diets were produced through extrusion, grinding, mixing, and pelleting. A low percentage of fish meal (20%) was used, resulting in a diet with 22% protein, close to the diets used by carp farmers in earthen ponds for the lowest possible cost. 

Analysis of the proximate composition of ingredients and diets was performed with a DA 7250 NIR Analyser (Perten Instruments, Hagersten, Sweden) (Table 2 and Table 3). The fish were distributed in 12 cylindrical fiberglass tanks with a volume of 0.75 m^3^. Each water tank housed 10 fish, resulting in 30 fish per experimental variant. 

In the beginning of the experiment, the fish were weighed and measured. Manual feeding with the experimental diets was performed three times a day (8:30 AM, 12:00 PM, 3:00 PM). The daily feed quantity was 3%, determined based on water temperature and fish weight.

### 2.3. Evaluation of Growth Performance

To determine the growth parameters, the fish were measured (in cm) and weighed (in g) three times during the indicated growth period. Based on these measurements, the main growth indices were determined and calculated, and were as follows:

- IBW (initial body weight, g);
- FBW (final body weight, g);
- WG (weight gain, g) = FBW − IBW;
- SGR (specific growth rate, % day^−1^) = (ln FBW − ln IBW)/days of experiment × 100;
- FCR (feed conversion ratio, g/g) = Feed intake (g)/WG;
- CF (condition factor) = FBW/body length^3^ × 100;
- PER (protein efficiency ratio) = WG/Protein intake (g);
- LER (lipid efficiency ratio) = WG/Lipid intake (g);
- HSI (hepatosomatic index, %) = 100 × [final liver weight (g)/final body weight (g)];
- VSI (viscerosomatic index, %) = 100 × [final visceral weight (g)/final body weight (g)].

### 2.4. Proximate Composition of Fish Meat

For the sampling procedure of fish flesh (muscle), the fish were euthanized using clove oil (2%). Nine samples from five fish were taken per variant to ensure accuracy and reliability in the analysis. The proximate composition analysis of the flesh was carried out using a DA 7250 NIR Analyzer manufactured by Perten Instruments in Hagersten, Sweden.

### 2.5. Blood Hematological Profile

Blood samples were collected from five fish per experimental variant after anesthetization in a 0.2% clove oil concentration for five minutes, using EDTA (ethylenediaminetetraacetic acid)-coated tubes. The evaluations were made using the hematology analyzer Abacus Vet 5, Budapest, Hungary (courtesy of Nova Group). 

### 2.6. Oxidative Status

After the feeding trial period ended, liver, muscle, and intestine tissue samples were precisely dissected and collected from four specimens for each experimental variant for oxidative status assessment. The tissue samples were homogenized in an ice-cold potassium phosphate buffer solution 0.1 M, KCl 1.15%, pH 7.4, in a ratio of 1:10 (*w*/*v*). The homogenates were centrifuged (20 min at 3000 rpm and 4 °C) and the supernatants were further used to measure the activities of superoxide dismutase (SOD), catalase (CAT), and glutathione peroxidase (GPX) and to determine the content of reduced glutathione (GSH) and malondialdehyde (MDA) according to the methods described in Barbacariu et al. [18]. SOD, CAT, and GPX activities and the levels of GSH and MDA were normalized to the total content of soluble proteins measured by the Bradford method [19].

### 2.7. Intestinal Microbiota

Intestine samples from five fish per variant were taken under laboratory conditions. After dissection, the intestinal contents were aseptically aspirated and 1 g of it was added to 9 mL of sterile saline solution using a sterile Pasteur pipette.

The quantitative determination of some groups of representative microorganisms in the gut microbiota of *Cyprinus carpio* fish was performed according to the methods described by Barbacariu et al. [20]. The microbiological indicators used in the quantitative assessment of the microbiota were as follows: the total number of aerobic bacteria (TNG-A), the total number of anaerobic bacteria (TNG-AN), the total number of sulfite-reducing *Clostridia* (TN-SRC), and the total number of *Enterobacteriaceae* (TN-E). The quantitative results of the microbiological tests were expressed in colony-forming units present in one gram of intestinal contents (CFU/g). To facilitate statistical analysis, the CFU/g values determined for each indicator were converted to decimal logarithms.

### 2.8. Statistical Analysis

Growth performance, flesh composition, blood biochemical profile, and intestinal microflora data were statistically processed by analysis of variance (ANOVA) followed by the Tukey test (*p* < 0.05) using SPSS software version 21 (IBM Corp, Armonk, NY, USA). Results are reported as means ± standard error of the mean (S.E.M.). Biochemical results (oxidative status) were analyzed by a two-way ANOVA followed by Tukey’s multiple comparisons tests using GraphPad Prism software v9.3.1 (La Jolla, CA, USA). Differences were considered significant when *p* < 0.05 and the values are expressed as means ± S.E.M.

## 3. Results

### 3.1. Evaluation of Growth Performance

The growth performance of carp is summarized in Table 4. The results demonstrate a clear trend in the growth performance of common carp in response to varying levels of sorghum inclusion in their diets. As the proportion of sorghum increased from V0 to V3, there was a noticeable decline in both final body weight (FBW) and weight gain (WG). Specifically, carp fed with the V3 diet containing 30% sorghum exhibited the lowest FBW and WG compared to those on other diets. However, no significant differences were observed in parameters such as feed conversion ratio (FCR), specific growth rate (SGR), condition factor (CF), protein efficiency ratio (PER), lipid efficiency ratio (LER), hepatosomatic index (HSI), and viscerosomatic index (VSI) across the experimental groups. These results indicate that while sorghum inclusion negatively impacted the growth performance of common carp in terms of final weight and weight gain, it did not significantly affect the other production traits measured in this study. Moreover, between the control group (V0) and the group with the lowest sorghum inclusion (V1), there were no significant differences observed in either FBW or WG. This suggests that the introduction of a small amount of sorghum (10%) into the diet did not markedly alter the growth performance of the common carp compared to the control group.

### 3.2. Proximate Composition of Fish Meat

The biochemical composition of common carp flesh fed with sorghum experimental diets is summarized in Table 5. Notable variations were observed in protein and salt content across the experimental groups. Protein content increased with higher levels of sorghum inclusion, reaching the highest value in the V2 group (17.00%). Conversely, salt content exhibited significant differences among the groups, peaking in the V2 group (3.74%). Moisture, fat, ash, and collagen content showed no significant variations among the experimental groups (*p* > 0.05). These results suggest that sorghum inclusion primarily influences protein and salt content in common carp flesh, potentially impacting its nutritional profile.

### 3.3. Results of Blood Hematological Examination

The blood parameters of common carp are presented in Table 6. White blood cell count (WBC) remained consistent across all groups, with no significant differences observed (*p* = 0.885). A slight lymphopenia associated with marked monocytosis and neutrophilia (doubling of the number of neutrophiles), suggestive for an inflammatory process due to sorghum inclusion, can be observed, however, these variations were not statistically significant. Eosinophil count (EOS) also exhibited slight variations across the groups, but these differences were not statistically significant (*p* = 0.297). Basophil count (BAS) and platelet count (PLT) remained stable across all groups, suggesting minimal impact on clotting function due to sorghum inclusion. Mean platelet volume (MPV) and platelets (PCT) did not exhibit significant differences between the groups, indicating consistent platelet size and volume across all dietary treatments.

### 3.4. Intestinal Microbiota

**Figure 1:**
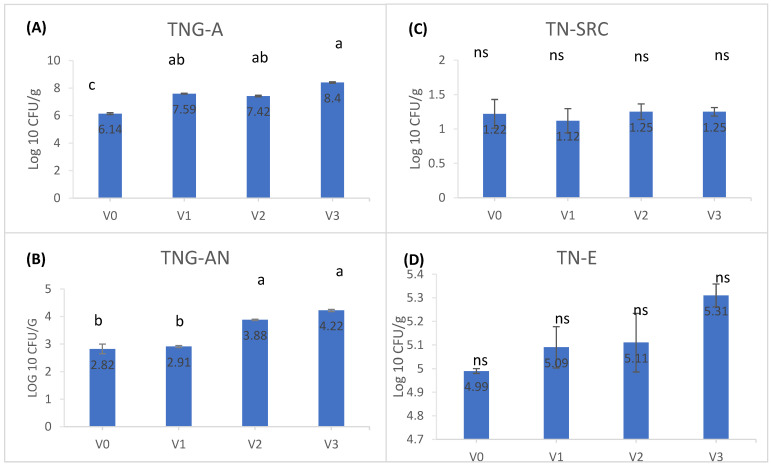
Graphical representation of the logarithm of the average number of CFU/g intestinal contents, for the indicators: (**A**) TNG-A (total number of aerobic bacteria), (**B**) TNG-AN (total number of anaerobic bacteria), (**C**) TN-SRC (total number of sulfite-reducing *Clostridia*), (**D**) TNE (total number of *Enterobacteriaceae*). Different lowercase letters represent statistically significant differences according to Tukey’s test at *p* < 0.05. ‘ns’ denotes non-significant differences.

The influence of sorghum experimental diets on the gut microbiota of carp is illustrated in Figure 1. Total aerobic bacteria (TNG-A) showed significant increases in V1, V2, and V3 compared to V0. Similarly, total anaerobic bacteria (TNG-AN) were notably higher in V2 and V3 compared to V0 and V1. No significant differences were observed in total sulfite-reducing clostridia (TN-SRC) and total enterobacteria (TN-E) across the groups (*p* > 0.05). The observed increases in both aerobic and anaerobic bacteria populations suggest a positive effect of sorghum inclusion on the gut microbiota of carp. This indicates that sorghum experimental diets may contribute to promoting a more diverse and potentially beneficial microbial community in the carp intestine.

### 3.5. Oxidative Stress

**Figure 2:**
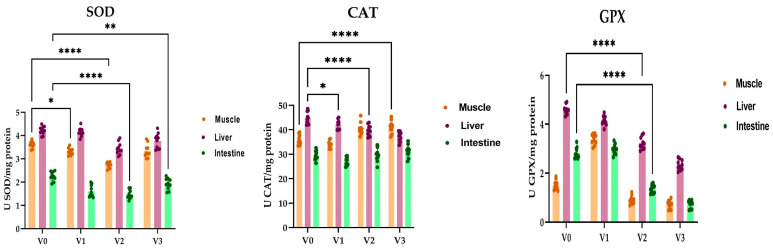
Sorghum influence on the oxidative status determined in muscle, liver, and intestine tissues of *Cyprinus carpio*. The enzymatic parameters consisted of measuring SOD, CAT, and GPX-specific activities. The values are expressed as means ± S.E.M. (n = 12). Two-way ANOVA analysis revealed overall significant differences between the experimental groups. For Tukey multiple comparisons analysis, **** *p* < 0.0001, ** *p* < 0.01, * *p* < 0.05.

The activity of the antioxidant enzymes SOD, CAT, and GPX is presented in Figure 2. The variation in enzyme activity in common carp is influenced by both tissue type and the concentration of sorghum in the experimental diets. As sorghum concentration increased, there were notable alterations in enzyme activities, particularly with decreasing trends observed in SOD activity, especially in the muscle (2.7 USOD/mg protein) and intestine (1.4 USOD/mg protein) tissue of V2 (*p* < 0.0001) compared to the control (3.6 USOD/mg protein in muscle and 2.2 USOD/mg protein in intestine). SOD activity decreased in liver tissue as well, from 4.2 USOD/mg protein in the control to 3.4 USOD/mg protein in V2; however, the differences were statistically insignificant. We observed that the lowest activity was detected in intestinal tissue, followed by muscle and liver tissues.

CAT activity in the fish liver decreased in V1 in comparison with the control group. We registered an increase in CAT activity in muscle tissue in V3 (44.8 UCAT/mg protein) compared with the control (39.7 UCAT/mg protein). In intestinal tissue, the variations were insignificant.

GPx in the liver of the control group was 4.5 UGPx/mg protein; the activity decreased in an inversely proportional relationship with the administered sorghum concentration (4.1 UGPx/mg protein in the case of the V1 variant, 3.2 UGPx/mg protein in the case of the V2 variant, and 2.3 UGPx/mg protein in the case of the group with 30% sorghum). The enzyme activity was almost identical in the samples taken from the intestinal tissue, the average value of GPx being 2.8 UGPx/mg protein in carp specimens from the control group and 2.9 UGPx/mg protein in V1. In the experimental variants where sorghum was administered in a concentration of 20 and 30%, the activity level reached thresholds of 1.3 UGPx/mg protein and 0.7 UGPx/mg protein. A completely different situation was observed at the muscle tissue level, where the highest average activity was recorded in V1 (3.3 UGPx/mg protein), while in variants V2 and V3, the activity of the enzyme was clearly inferior to that in both the control group (0.9 UGPx/mg protein in V2 and 0.7 UGPx/mg protein in V3, compared to 1.5 UGPx/mg protein in the control) and in V1.

**Figure 3:**
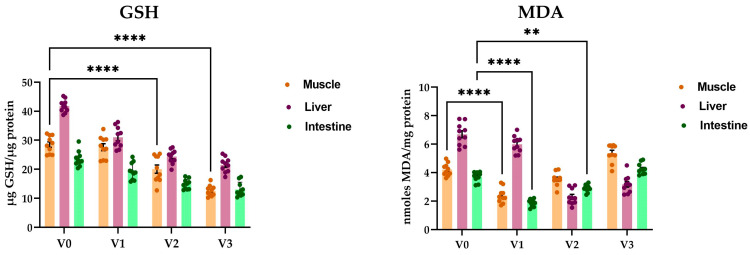
Sorghum influence on the oxidative status determined in muscle, liver, and intestine tissues of *Cyprinus carpio*. The non-enzymatic parameters consisted of estimating the levels of GSH and MDA. The values are expressed as means ± S.E.M. (n = 12). Two-way ANOVA analysis revealed overall significant differences between the experimental groups. For Tukey multiple comparisons analysis, **** *p* < 0.0001, ** *p* < 0.01.

The concentration of GSH in carp decreased in correlation with sorghum concentration in the diet (Figure 3). In the control variant, GSH values were 28.5 µg GSH/µg protein in the muscle, 41.7 µg GSH/µg protein in the liver, and 23.6 µg GSH/µg protein in the intestine. However, in variant V2 with 20% sorghum, average values dropped to 20.0 µg GSH/µg protein in the muscle, 24.3 µg GSH/µg protein in the liver, and 14.8 µg GSH/µg protein in the intestine. Notably, the group treated with 30% sorghum exhibited significantly lower GSH concentrations across all tissue types, with average values halved compared to the control group: 12.6 µg GSH/µg protein in the muscle, 21.5 µg GSH/µg protein in the liver, and 13.0 µg GSH/µg protein in the intestine.

Sorghum administration in carp diets had varied effects on MDA content in the analyzed tissues (Figure 3). In muscle tissue, the average MDA concentration in the reference group was 4.2 nmoles MDA/mg protein, decreasing in experimental variants V1 (2.3 nmoles MDA/mg protein) and V2 (3.5 nmoles MDA/mg protein). However, in carp fed with 30% sorghum, MDA levels were 1.27 times higher than in the control (5.3 nmoles MDA/mg protein). A similar trend was observed in intestinal tissue, with maximum MDA recorded in the control group (3.7 nmoles MDA/mg protein) and decreasing in the 10% sorghum concentration group (1.8 nmoles MDA/mg protein). Liver tissue exhibited the highest MDA concentrations, with the control and V1 variants showing maximum average values of 6.6 nmoles MDA/mg protein and 5.9 nmoles MDA/mg protein, respectively. Notably, the V2 variant displayed the lowest value, at 2.2 nmoles MDA/mg protein.

The experimental findings demonstrate a clear association between sorghum concentration in carp diets and alterations in both glutathione (GSH) concentration and malondialdehyde (MDA) content across different tissue types. As sorghum concentration increased, there was a significant decrease in GSH levels, indicating a potential disruption in antioxidant defense mechanisms, while MDA levels concurrently increased, suggesting elevated oxidative stress levels.

## 4. Discussion

The inclusion of sorghum in carp feed formulations at concentrations of 20% and 30% resulted in significant reductions in final body weight and weight gain. However, at 10% sorghum inclusion, these parameters were not significantly affected. Furthermore, weight gain, feed conversion ratio, and condition factor were not affected in any of the variants, even when corn and barley were completely replaced by sorghum at 30%. These findings are supported by the proximate composition of sorghum, which revealed a protein content of 14.31%, fat content of 3.97%, and starch content of 50.5%. This suggests that sorghum can be a viable and sustainable alternative in enhancing the efficiency and cost-effectiveness of carp farming operations. Based on the current study, sorghum is effective when comprising a total of 10% of the diet. Sorghum protein content varies based on environmental conditions, growing region, and soil type, ranging from 8 to 15% on a dry matter basis [2]. It can be classified into various soluble fractions, such as albumins, globulins, prolamins, kafirins, cross-linked kafirins, cross-linked glutelins, and unextracted protein residue [2]. Despite the diversity in amino acid profiles among sorghum varieties, sorghum proteins, like other plant proteins, tend to be lower in essential amino acids such as lysine, tryptophan, and threonine compared to animal-based ingredients [2]. While limited data are available, they suggest that carp, tilapia, and silver catfish can digest and metabolize sorghum-based diets [2,16,21]. In carp, the protein digestibility of sorghum was 71.86%, while fat digestibility was 76.71% [16]. In silver catfish (*Rhamdia quelen*), the inclusion of sorghum in diet led to increased FCR, likely linked to its adverse effects on nutrient absorption in the fish. The predominant protein in sorghum is kafirin, which exhibits a rigid structural configuration within the starch protein matrix, low solubility, and an imbalanced amino acid profile, contributing to reduced food digestibility [21]. The most common carp farming technology is in earthen ponds, in a semi-intensive system. In these systems, feeding is conducted using either pelleted feeds or a ground mixture of various ingredients, primarily cereals, but also animal- and plant-origin flours and meals. As feeding represents over 50% of production costs, identifying more sustainable ingredients is timely. While for super-intensive systems, the FCR (feed conversion ratio) can reach a value of 1, in semi-intensive systems such as those for carp, the FCR can reach values of 3.5 [22]; thus, creating cheap recipes with new ingredients and lower FCRs is of interest.

The proximate composition of fish meat, including moisture, protein, fat, ash, collagen, and salt content, reflects its nutritional quality and consumer appeal. While moisture content showed minimal variation among treatment groups (68.74% to 69.86%), significant differences were observed in protein levels, with the highest content in V2 (17%) and the lowest in the control group (15.64%), indicating the potential of dietary interventions to influence protein deposition in fish. These findings highlight the potential of dietary interventions to influence fish meat composition, impacting its sensory and nutritional properties. Additionally, Hussein et al. (2016) [23] found an increase in protein content in the meat of Nile tilapia (*Oreochromis niloticus*), while Obe (2014) [24] observed a similar increase in the meat of a catfish hybrid (*Heterobranchus bidorsalis* × *Clarias gariepinus*). Sorghum was the studied ingredient used in the diets in the previously mentioned studies.

Various factors, including diet composition, environmental conditions, and physiological status, can significantly influence hematological parameters in fish [25,26]. Leukocyte count (WBC) is an important parameter in the assessment of immune status in vertebrates, encompassing various cell types such as lymphocytes, monocytes, neutrophils, eosinophils, and basophils. In our study, leukocyte count notably decreased in the sorghum-treated variants, particularly in V2 and V3. Interestingly, monocyte levels increased in V2, indicating a potential inflammatory response. The cause of these variations may be represented by the effect of microbiota changes due to the action of feed compounds, favoring the multiplication of some species to the detriment of others, causing a dysmicrobism, with effects on cell populations. Other factors can be related to environmental parameters.

The gut microbiome of fish is extremely complex, consisting of aerobic, facultative anaerobic, and obligate anaerobic microorganisms varying between 107 and 1011 cells/gram of gut contents, according to some authors [27,28]. The microbiological indicators determined in this study, used as a quantitative benchmark in the evaluation of the effect induced by sorghum nutrients on the gut microbiota, are the main colonizers of the gastrointestinal tract of fish. Of course, the gut microflora varies depending on the complexity of the fish’s digestive system, aquatic habitat, aquatic microflora, and feeding technology [29,30]. More and more studies show that the diet used in fish nutrition is related to the composition of the gut microbiome [31], which is closely related to the immune system, and together they are responsible for the overall health status. In the experimental conditions of our study, the results of the microbiological indicators in the experimental groups fed diets supplemented with 10% (V1), 20% (V2), and 30% (V3) sorghum were found to exceed the results of the control group fed the basal diet. The analysis of the four microbiological indicators clearly showed the dominance of aerobic/facultative anaerobic microbiota compared to strictly anaerobic microbiota in the intestine of *Cyprinus carpio*. A comparison of these results with those of the control group shows that the diet supplemented with different concentrations of sorghum provides favorable nutrients for the maintenance and even proliferation of commensal bacteria in the digestive tract. The presence of anaerobic bacteria in the digestive tract of fish is crucial for maintaining a balanced microbial ecosystem, and they have been frequently detected in the guts of carp and tilapia [29,31]. This anaerobic microbiota also include sulfite-reducing Clostridia, which are representative of monitoring water hygiene [32] and are of major public health concern when they contaminate food [33]. However, we can consider a balanced diet that favors a moderate proliferation of bacterial populations in the digestive tube of carp to be beneficial, as it increases protection against digestive diseases, given the known role of commensal microorganisms in the host’s defense against transient pathogenic and nutrient-hungry microorganisms in the digestive tube [34].

Oxidative stress, the imbalance between the production of reactive oxygen species (ROS) and antioxidant defenses, can alter the structure and the function of mitochondrial and cellular components [35]. In fact, the term oxidative stress relates to an excess of pro-oxidative factors (ROS) over antioxidants. Due to having an unpaired electron, free radicals are unstable and highly reactive. A free radical reacts with susceptible compounds, including lipids, proteins, and/or DNA. All these reactions result in the formation of a new free radical, excessive concentrations of ROS causing major oxidative damage [36]. This so-called oxidative damage can have a pronounced impact on fitness by reducing fertility and accelerating ageing. Oxidative stress has been linked to many pathologies; these associations emphasize that a balance must be found between the relative abundance of ROS and antioxidants. Cells possess complex biochemical and genetic mechanisms to maintain such a balance, and it is clear that their disruption can have profound pathophysiological consequences [37]. In order to avoid excessive levels of oxidative damage following an increase in ROS production, organisms can increase their synthesis of endogenous antioxidant enzymes (superoxide dismutase, catalase, glutathione peroxidase) to counteract the action of ROS or replace molecular constituents susceptible to the action of ROS (e.g., polyunsaturated fatty acids) with less sensitive ones (e.g., monounsaturated fatty acids) [38,39].

Data in the literature show that sorghum has comparative nutritional value to other cereals in terms of chemical composition, protein, fat, carbohydrates, and non-starch polysaccharides, as well as bioactive components like vitamin B and fat-soluble vitamins (D, E, and K), dietary fibers, policosanols, iron, zinc, copper and manganese, tocols, phytosterols, γ-oryzanol, phytic acid, micronutrients, macronutrients, essential minerals, and non-nutrients like carotenoids and polyphenols [40,41]. Sorghum varieties are rich in bioactive phenolic compounds [41] such as ferulic acid, gallic acid, vanillic acid, caffeic acid, cinnamic acid, salicylic acid, p-coumarinic acid, luteolin, apigenin, and 3-deoxyanthocyanidins. These constituents contribute to the numerous health benefits of this grain, including anti-oxidative, anti-inflammatory, anti-proliferative, anti-diabetic, anti-atherogenic activities, antimicrobial properties, and anti-cancer effects, as well as free radical scavenging and high antioxidant activity [42,43,44,45,46]. Tannins in sorghum impact the availability of minerals, proteins, and starch. Due to their significant interaction with starch and particularly amylose, polymeric tannins are key in producing resistant starch. The tannin oligomers present human health benefits through their immunomodulatory, antiradical, vasodilatory, cardioprotective, antithrombotic, and anti-UV actions [47,48,49]. Others potential health benefits are risk reduction for obesity, dyslipidemia, hypertension, and promoting urination and blood circulation [50]. Due to these properties, but also to its agronomic advantages (high yield, drought tolerance, and low production costs), interest in sorghum for human and animal consumption has increased considerably worldwide [51]. In addition, sorghum stands out compared to other grains due to its high antioxidant activity [52].

Our experimental results showed wide variations in superoxide dismutase (SOD) activity depending on the investigated tissue and the experimental variant. While the control group exhibited SOD activity levels of 3.6 USOD/mg protein in muscle, 4.2 USOD/mg protein in liver tissue, and 2.2 USOD/mg protein in intestinal samples, a decrease in enzyme activity was observed in direct correlation with the added sorghum concentration in experimental variants, particularly in variant V2 with 20% sorghum. For variant V2, activity thresholds of 1.4 USOD/mg protein in the intestine, 2.7 USOD/mg protein in muscle, and 3.4USOD/mg protein in liver tissue were recorded. Superoxide dismutase is a critical enzyme in the body’s first line of defense, an important and indispensable marker in the entire antioxidant defense strategy, especially in reference to the superoxide anion radical (*O2), which is perpetually generated in normal body metabolism, especially through the mitochondrial energy production pathway [53].

Our results also highlighted a decrease in CAT activity in fish liver in all experimental variants compared to the reference group, while a slight increase in activity was observed in muscle and intestinal tissue in variants with sorghum. In another series of experiments, we determined the activity of catalase, an enzyme that plays a crucial role in the adaptive response to H_2_O_2_ [54], suggesting that its role is somehow auxiliary to that of glutathione peroxidase [55]. Using radio-labeling and aminotriazole inhibition, it was shown that CAT takes care of 50% of the hydrogen peroxide, and glutathione peroxidase/reductases take care of the other half [56]. Moreover, the literature data indicate increased levels of the enzyme, obtained by overexpression in different organelles, which extended the lifespan of transgenic laboratory mice by 20%, the greatest increase being related to the mitochondria, where the highest levels of reactive oxygen species are generated [56,57,58,59].

The situation is different in the case of glutathione-peroxidase, an important cellular antioxidant enzyme found in the cytoplasm and mitochondria of eukaryotic cells that modulates the balance between necessary and harmful levels of reactive oxygen species, using glutathione (GSH) as a reducing agent. The GPx enzyme exhibited a completely different behavior in carp specimens from experimental variants, showing a close dependence on the type of tissue analyzed and the concentration of sorghum added to the diet. GSH concentration in *Cyprinus carpio* L. specimens decreased in correlation with the concentration of sorghum in the diet, with the lowest concentrations observed in the group treated with 30% sorghum across all tissue types analyzed. The literature data emphasizes that muscular exertion can cause an imbalance between prooxidants and antioxidants in skeletal muscle, with oxygen flow being able to increase considerably during powerful exercise, while in fish antioxidant enzyme activities appear to depend on mainly by their swimming ability and lifestyle rather than evolutionary group [60]. An important component of the antioxidant defense system in fish is glutathione (GSH), a tripeptide that directly or indirectly regulates the elimination of ROS and their reaction products [61,62], through a cumulative action with several other associated enzymes, such as GSH-peroxidase (EC 1.11.1.9), GSH-reductase (EC 1.6.4.2), and Glutathione-S-transferase (EC 2.5.1.18). The literature in the field highlights the importance of fish nutrition, as the enrichment of food with antioxidants is able to improve resistance to various stress conditions, increasing antioxidant capacity and ensuring superior qualities of aquaculture products [63].

In the final stage of our experiment, we determined the concentration of MDA, a small and reactive organic molecule that occurs ubiquitously among eukaryotes, formed by three carbon molecules with two aldehyde groups at the carbon 1 and carbon 3 positions. The measurement of malondialdehyde content has long been used as a lipid peroxidation marker in studies related to oxidative stress and redox signaling [64]. The administration of sorghum had varied influences on the content of MDA in the analyzed biological tissues, with significant changes observed in muscle, liver, and intestinal tissue samples. Scientific data indicate the significant influence that dietary supplementation with different agents has on the concentration of MDA in different tissues and organs, the highest values being found, as a rule, in the liver [65,66]. It should also be noted that sorghum is a grain rich in protein, lipids, and, above all, carbohydrates; the literature data emphasize that a diet rich in carbohydrates and lipids can cause a significant decrease in the concentration of MDA in fish and, implicitly, increase their physiological condition [41,67].

## 5. Conclusions

This study highlights the significant impact of including sorghum in the common carp’s diets on various physiological parameters. While sorghum positively influenced protein and salt content in fish flesh and promoted intestinal microbiota diversity, it adversely affected growth performance and oxidative stress indicators. Decreased glutathione levels and increased malondialdehyde content suggest compromised antioxidant defense mechanisms and elevated oxidative stress levels with higher sorghum concentrations. These findings underscore the importance of carefully considering dietary composition in fish nutrition to optimize growth performance and overall health. Notably, at a 10% sorghum inclusion level, no significant effects on final body weight and weight gain were observed, suggesting a potential threshold where the detrimental impacts on growth performance may be mitigated.

## Figures and Tables

**Table 1:** Water quality in the RAS during the fish trial with experimental sorghum diets.

Parameter	Units	Week 1	Week 2	Week 3	Week 4	Week 5	Week 6
Temperature	°C	25.60	22.90	25.00	25.10	23.00	21.70
pH	units	8.10	7.79	7.88	7.77	7.42	7.86
Dissolved oxygen	mg/L	8.00	8.10	8.00	8.00	8.10	8.00
Nitrate (NO₃^−^)	mg/L	0.22	0.16	0.10	0.02	0.07	0.04
Nitrite (NO₂^−^)	mg/L	0.28	0.14	0.10	0.02	0.10	0.03
Ammonia (NH₃⁺)	mg/L	0.05	0.12	0.06	0.02	0.01	0.20
Ammonium (NH₄⁺)	mg/L	12.60	14.40	11.00	10.00	12.00	10.00
Phosphorus total (PO₄^3−^)	mg/L	7.80	8.00	8.10	7.80	7.70	8.00
Hardness total	°G	98.00	95.00	100.00	84.00	88.00	96.00
Calcium (Ca₂⁺)	mg/l	54.00	66.00	64.00	61.00	57.00	59.00
Magnesium (Mg₂⁺)	mg/l	0.00	0.00	0.00	0.00	0.00	0.00
Iron (Fe)	mg/l	116.00	128.00	122.00	100.00	112.00	124.00
Sulfate (SO₄^2−^)	mg/l	0.10	0.00	0.10	0.06	0.00	0.10

**Table 2:** Proximate composition of the ingredients used in experimental sorghum diets for carp.

Ingredient (%)	Moisture	Protein	Fat	Fiber	Starch	Ash	Sugar	Calcium	Phosphorus
Sorghum	13.17	14.31	3.97	4.55	50.50	12.30	2.59	1.06	0.68
Sunflower meal	10.46	41.10	1.63	22.10	-	7.54	-	-	-
Green peas	11.2	22.35	-	-	-	-	-	-	-
Corn	12.34	7.51	3.60	-	63.11	-	-	-	-
Barley	11.29	11.78	-	4.97	53.20	-	-	-	-
Corn DDGS	10.47	26.67	-	9.48	3.72	3.96	-	-	0.69
Fish meal	10.09	36.50	8.40	-	-	24.40	-	-	-

**Table 3:** Ingredients and proximate composition of experimental diets for carp.

Ingredient (%)	V0 (0% Sorghum)	V1 (10% Sorghum)	V2 (20% Sorghum)	V3 (30% Sorghum)
Sunflower meal	20	20	20	23
Green peas	20	20	20	17
Corn	15	13	8	-
Barley	12	5	-	-
Corn DDGS	11	11	11	10
Fish meal	20	20	20	20
Sunflower oil	2	1	1	-
Sorghum	-	10	20	30
**Proximate composition (%)**				
Moisture	9.71 ± 0.35	10.16 ± 0.10	10.42 ± 0.18	10.34 ± 0.26
Protein	22.34 ± 0.24	22.17 ± 0.12	22.07 ± 0.15	22.48 ± 0.19
Fat	5.30 ± 0.08	5.20 ± 0.04	5.74 ± 0.03	5.05 ± 0.02
Fiber	6.66 ± 0.07	6.33 ± 0.01	5.99 ± 0.05	6.43 ± 0.01
Starch	60.10 ± 0.26	58.87 ± 0.07	58.47 ± 0.28	57.43 ± 0.23
Ash	8.63 ± 0.12	8.17 ± 0.07	8.63 ± 0.09	8.97 ± 0.07
Sugar	2.90 ± 0.15	2.90 ± 0.04	2.89 ± 0.09	2.22 ± 0.07
Phosphorus	0.06 ± 0.02	0.07 ± 0.01	0.10 ± 0.03	0.08 ± 0.01

**Table 4:** Growth performance parameters of common carp fed sorghum experimental diets.

Parameter	V0 (0% Sorghum)	V1 (10% Sorghum)	V2 (20% Sorghum)	V3 (30% Sorghum)	ANOVA
IBW (g)	44.39 ± 1.33 ns	43.6 ± 0.92 ns	43.01 ± 1.26 ns	41.72 ± 1.44 ns	0.49
FBW (g)	76.54 ± 1.81 a	70.71 ± 1.52 ab	66.48 ± 1.74 b	65.46 ± 2.49 b	0.00
WG (g)	32.05 ± 2.26 a	26.8 ± 0.47 ab	23.07 ± 1.01 b	24.47 ± 1.36 b	0.01
FCR (g/g)	2.91 ± 0.49 ns	2.99 ± 0.45 ns	3.13 ± 0.39 ns	3.18 ± 0.37 ns	0.969
SGR (% day^−1^)	1.95 ± 0.19 ns	1.71 ± 0.03 ns	1.53 ± 0.02 ns	1.65 ± 0.12 ns	0.133
CF	2.29 ± 0.27 ns	1.94 ± 0.03 ns	1.96 ± 0.03 ns	1.95 ± 0.03 ns	0.214
PER (g/g)	1.94 ± 0.33 ns	1.78 ± 0.27 ns	1.65 ± 0.21 ns	1.51 ± 0.22 ns	0.699
LER (g/)	7.56 ± 1.3 ns	8.97 ± 1.36 ns	7.34 ± 0.95 ns	8.41 ± 1.22 ns	0.763
HSI (%)	0.69 ± 0.08 ns	0.8 ± 0.05 ns	0.75 ± 0.03 ns	0.77 ± 0.03 ns	0.525
VSI (%)	15.99 ± 2.51 ns	17.14 ± 3.03 ns	20.11 ± 4.3 ns	22.28 ± 3.11 ns	0.555

IBW—initial body weight, FBW—final body weight, WG—weight gain, FCR—feed conversion ratio, CF—condition factor, PER—protein efficiency ratio, LER—lipid efficiency ratio, HSI—hepatosomatic index, and VSI—viscerosomatic index. Different lowercase letters represent statistically significant differences according to Tukey’s test at *p* < 0.05. ‘ns’ denotes non-significant differences.

**Table 5:** Biochemical composition of common carp flesh fed sorghum experimental diets.

Parameter (%)	V0 (0% Sorghum)	V1 (10% Sorghum)	V2 (20% Sorghum)	V3 (30% Sorghum)	ANOVA
Moisture	69.12 ± 0.51 ns	69.86 ± 0.33 ns	68.74 ± 0.88 ns	69.12 ± 0.59 ns	0.630
Protein	15.64 ± 0.31 b	16.22 ± 0.24 ab	17.00 ± 0.25 a	15.98 ± 0.16 b	0.008
Fat	6.28 ± 0.53 ns	5.32 ± 0.58 ns	6.56 ± 1.24 ns	5.60 ± 1.34 ns	0.798
Ash	2.76 ± 0.17 ns	3.14 ± 0.2 ns	3.14 ± 0.16 ns	3.04 ± 0.16 ns	0.392
Collagen	1.06 ± 0.09 ns	0.78 ± 0.15 ns	0.70 ± 0.28 ns	0.84 ± 0.13 ns	0.540
Salt	2.34 ± 0.12 b	3.46 ± 0.43 ab	3.74 ± 0.37 a	2.82 ± 0.34 ab	0.041

Different lowercase letters represent statistically significant differences according to Tukey’s test at *p* < 0.05. ‘ns’ denotes non-significant differences.

**Table 6:** Effect of experimental diets with sorghum on hematological parameters in common carp.

Parameter	V0 (0% Sorghum)	V1 (10% Sorghum)	V2 (20% Sorghum)	V3 (30% Sorghum)	ANOVA
WBC (X10^9^/l)	49.3 ± 2.36 ns	50.92 ± 2.56 ns	48.76 ± 4.42 ns	47.47 ± 2.64 ns	0.885
LYM (X10^9^/l)	44.41 ± 2.02 ns	38.84 ± 1.50 ns	40.91 ± 6.08 ns	41.34 ± 5.69 ns	0.839
MON (X10^9^/l)	0.37 ± 0.10 ns	3.96 ± 0.27 ns	3.74 ± 1.29 ns	1.61 ± 1.36 ns	0.077
NEU (X10^9^/l)	6.25 ± 1.48 ns	8.12 ± 0.80 ns	4.12 ± 2.26 ns	4.52 ± 1.84 ns	0.376
EOS (X10^9^/l)	0.81 ± 0.21 ns	0.61 ± 0.12 ns	0.48 ± 0.04 ns	0.49 ± 0.08 ns	0.297
BAS (X10^9^/l)	0.04 ± 0.01 ns	0.03 ± 0.01 ns	0.02 ± 0.01 ns	0.02 ± 0.01 ns	0.524
PLT (X10^9^/l)	42.33 ± 1.33 ns	45.33 ± 5.46 ns	45.33 ± 5.70 ns	34.00 ± 7.37 ns	0.453
MPV (fl)	9.33 ± 0.07 ns	9.4 ± 0.12 ns	9.67 ± 0.52 ns	9.40 ± 0.32 ns	0.878
PCT (%)	0.04 ± 0.00 ns	0.04 ± 0.00 ns	0.05 ± 0.01 ns	0.03 ± 0.01 ns	0.181

WBC (white blood cells), LYM (lymphocytes), MON (monocytes), NEU (neutrophils), EOS (eosinophils), BAS (basophils), PLT (platelets), MPV (mean platelet volume), PCT (plateletcrit). ‘ns’ denotes non-significant differences.

## Data Availability

All data generated by this study are present in this article.
